# Comparison of Microwave Hyperthermia Applicator Designs with Fora Dipole and Connected Array

**DOI:** 10.3390/s23146592

**Published:** 2023-07-21

**Authors:** Gulsah Yildiz, Iman Farhat, Lourdes Farrugia, Julian Bonello, Kristian Zarb-Adami, Charles V. Sammut, Tuba Yilmaz, Ibrahim Akduman

**Affiliations:** 1 Department of Electronics and Communication Engineering, Istanbul Technical University, 34469 Istanbul, Turkey; 2 Department of Physics, University of Malta, MSD 2080 Msida, Malta; 3 Mitos Medical Technologies, 34467 Istanbul, Turkey

**Keywords:** microwave hyperthermia, cancer therapeutics, fractal octagonal ring array, dipole antenna, connected array, particle swarm optimization

## Abstract

In microwave hyperthermia tumor therapy, electromagnetic waves focus energy on the tumor to elevate the temperature above its normal levels with minimal injury to the surrounding healthy tissue. Microwave hyperthermia applicator design is important for the effectiveness of the therapy and the feasibility of real-time application. In this study, the potential of using fractal octagonal ring antenna elements as a dipole antenna array and as a connected array at 2.45 GHz for breast tumor hyperthermia application was investigated. Microwave hyperthermia treatment models consisting of different fractal octagonal ring antenna array designs and a breast phantom are simulated in COMSOL Multiphysics to obtain the field distributions. The antenna excitation phases and magnitudes are optimized using the global particle swarm algorithm to selectively increase the specific absorption rate at the target region while minimizing hot spots in other regions within the breast. Specific absorption rate distributions, obtained inside the phantom, are analyzed for each proposed microwave hyperthermia applicator design. The dipole fractal octagonal ring antenna arrays are comparatively assessed for three different designs: circular, linear, and Cross—array. The 16-antenna dipole array performance was superior for all three 1-layer applicator designs, and no distinct difference was found between 16-antenna circular, linear, or cross arrays. Two-layer dipole arrays have better performance in the deep-tissue targets than one-layer arrays. The performance of the connected array with a higher number of layers exceeds the performance of the dipole arrays in the superficial regions, while they are comparable for deep regions of the breast. The 1-layer 12-antenna circular FORA dipole array feasibility as a microwave hyperthermia applicator was experimentally shown.

## 1. Introduction

Hyperthermia (HT) studies have attested that elevated tissue temperature can damage and shrink cancerous cells while causing minimal harm to normal tissues [1]. Moreover, HT makes some of the tumor cells more susceptible to radiation therapy and chemotherapy [2]. Hence, it has been used to treat different types of advanced cancers in combination with various other forms of cancer therapy, including radiation therapy and chemotherapy [3]. HT is a process that artificially elevates tumor temperature to between 40 °C and 45 °C for a sufficient period of time (30–60 min) while maintaining the normal body temperature in the remaining tissue [3]. HT can be delivered using three different modalities, such as ultrasound, thermal conduction, and microwave radiation devices. However, the final effect varies for every tissue depending on its location and constituents, such as fat, water, or bone. This study focuses on a noninvasive system of microwave hyperthermia (HT) to be used in the treatment of breast cancer.

A relatively high proportion of carcinomas arises in the upper outer quadrant of the breasts [4]. However, malignant tissues can develop deep or at any location within the breast, even near the chest wall. Hence, clinical HT applicators should possess controllable power deposition profiles to treat lesions of varying sizes and shapes that may occur at diverse locations within the breast. HT employs beamforming techniques to focus microwave energy on the breast tumor by adjusting the excitation phase and amplitudes of the antenna array. Therefore, the choice of the HT applicator and its array distribution is very important in hyperthermia treatment planning (HTP). Circular-shaped applicators (CA) are the most common applicator type in breast HT [5,6,7]. The symmetry offered by CA provides good coverage of the breast tissue. Linearly distributed applicator (LA) designs have also been proposed [8,9,10]; however, their practice has not been appealing because it has low coverage due to non-symmetrical geometry. In a recent study, the physical rotation of the linear array has been proposed to better align LA with the target, and thus enhance the focusing performance [11]. Furthermore, a formation of a hemispherical hyperthermia chamber deploying an antenna array around the breast has been shown to realize selective heating of a tumor in a sample [12,13,14]. It offers a comfortable and wearable hyperthermia system that can provide complete coverage of the breast. This enables conformal tumor heating by mechanically or electronically scanning a highly focused beam through the target region at various breast locations [12,15]. Moreover, efficient HT focusing can be achieved for small deep-seated breast tumors utilizing radiating elements implemented in a symmetric cross configuration [16,17]. In [18,19] a Cross—array (XA) arrangement of four sub-arrays of corrugated tapered slot antennas for application in three-dimensional (3D) HT was investigated using MRI-derived realistic 3D breast phantoms in full-wave electromagnetic simulations. The results of this array configuration illustrated the possibility of selectively heating a tumor volume of 1 cm${}^{3}$ in gland tissue.

This paper employs fractal octagonal ring antenna (FORA) elements for a near-field phased array antenna and explores its feasibility for an efficient HT system. FORA is a printable antenna that can be tailored for a wide range of applications [20]. Implementation of FORA under flat and curvature conditions would be valuable for HT therapy systems to follow the patient’s body contour. Two types of antenna system were adopted using FORA elements, which are the disconnected dipole arrays and the connected arrays. The FORA dipole array is a standard narrow-band antenna design, intended to keep low mutual coupling between the radiating elements, so as not to unduly alter the performance of each isolated element. On the other hand, a FORA—connected array is a broadband array design in which mutual coupling is intentionally introduced between the array elements in addition to capacitance coupling between the tip ends of each element [21]. This enables almost continuous current flow among the different FORA elements, thus realizing the continuous current sheet proposed by Wheeler [22]. The earlier works of the authors are related to wide-band Vivaldi antennas, tailored especially for imaging applications [8,10,11]. In this work, FORA dipole provides the narrow-band application, while FORA—connected array provides the conformal and wide-band characteristic that can be used in a future wearable hyperthermia device application.

For efficient microwave focusing, global particle swarm optimization (PSO) was used to find the optimum antenna excitations, which enables constructive interference in the desired target region and destructive interference elsewhere. To computationally verify the FORA array performance, a 3D simulation of a cylindrical breast phantom was performed, and two-dimensional (2D) optimization of the antenna excitation parameters was conducted, then the SAR and temperature results were represented in 2D planes.

FORA dipoles were arranged in circular, linear, and Cross—array applicator designs which are compatible with the cylindrical phantom geometry. To assess the applicator design performance in a simple medium, a homogeneous fat phantom was used, and the positions of different applicator designs were pre-adjusted to show their best performance at the investigated target locations. One-layer dipole applicators were investigated for varying antenna numbers and inter-antenna distances. The best-performing designs of the 1-layer applicators were then investigated for two layers, and for the inter-layer distance. The FORA—connected array was investigated for different antenna numbers and layer numbers, and thus different curvatures. Comparisons between the 1- and 2-layer FORA dipole applicators and different designs of FORA—connected arrays are given. Comparisons presented in this paper are based on the target-to-breast SAR ratio and the necessary total antenna power to reach the desired temperature at the target.

An experimental setup with a 1-layer 12-antenna circular FORA dipole array and fat-mimicking phantom is presented. The computed result and the experimental result are compared to each other.

The main contributions of this work can be summarized as follows:

- FORA antenna element is proposed to be used in an HT application.
- FORA element is comparatively assessed in the forms of dipole and connected arrays.
- The FORA dipole arrays are comparatively assessed for three different designs (circular, linear, and Cross—array applicators); the number of constituent antennas; the number of antenna layers; inter-antenna distances; antenna-tissue distances; and inter-layer distances.
- The FORA—connected arrays are comparatively assessed for the number of constituent antennas; number of antenna layers; and antenna-tissue distances.
- The use of the FORA circular array as a hyperthermia applicator was experimentally verified on a fat-mimicking phantom.

The rest of the paper is organized as follows: an introduction to Pennes’ bio-heat equation that governs the thermodynamic relation in space and time between the SAR and the tissue temperature is given in Section 1.1. The following methodology is provided in Section 2, where the phantom, the FORA antenna, the HT applicator designs, and the simulation environment are explained in detail. In Section 3, 1- and 2-layer dipole HT applicator design results as well as the connected FORA array design results are provided, and the experimental results are presented. Finally, the authors conclude the work in Section 4.

### 1.1. Pennes’ Bio-Heat Equation

The normal body tissue temperature is $T_{0}=37{\;}^{\circ }C$. Once heated with an applicator during hyperthermia therapy, the heat transmission process in living tissue includes thermal conduction, blood circulation and perfusion, and metabolic heat output. Pennes [23] was the first to develop a mathematical model that describes heat transfer in human tissue involving the effects of blood flow on tissue temperature on a continuum basis, assuming the venous blood temperature is equivalent to the local tissue temperature. Pennes’ bio-heat equation is the most widely used thermal model for studying heat transfer phenomena associated with hyperthermia treatment modalities. For the transient problem, the temperature distribution in the breast phantom is addressed by Pennes’ bio-heat model expressed in (1), which allows for a different blood temperature.

(1) $$\begin{array}{c} C_{p}\rho \frac{\partial T}{\partial t}=\nabla \cdot \left(K\nabla T\right)+Q_{m}+\rho SAR-B\left(T-T_{b}\right) \end{array}$$

where $C_{p}$ is tissue-specific heat capacity, ρ is the tissue density, *K* is thermal conductivity, *T* is the temperature, $T_{b}$ is the blood temperature, $Q_{m}$ is the metabolic heat generation, *B* is the capillary blood perfusion coefficient. These parameters are tissue-specific. The specific absorption rate (SAR) depends on the external heating source, as well as tissue-specific parameters. SAR can be formulated as:

(2) $$\begin{array}{c} SAR=0.5\frac{{\sigma |E|}^{2}}{\rho }\;W/kg \end{array}$$

where *E* is the electric field (V/m) in the tissue and σ (S/m) is the electrical conductivity. By Green’s function approach, it was shown in [5] that the maxima of SAR and the temperature are located at the same point, assuming *K* and *B* are constants and in a steady state. Based on this result, the approach adopted in the present work was first to focus the maximum SAR on the target tissue and then scale the intensity to reach the desired tissue temperature.

## 2. Materials and Methods

### 2.1. Numerical Breast Phantom

We used a numerical homogeneous breast phantom model centered at (0, 0, 0) mm, consisting of fatty breast tissue in the form of a cylinder with a diameter and height of 90 mm. The phantom dielectric properties were taken from the phantom repository of the University of Wisconsin Cross-Disciplinary Electromagnetics Laboratory [24,25]. Fatty-1 tissue was used for the fat cylinder phantom, and the Debye parameters provided by [24,25] are given in Table 1. The constant dielectric parameters were calculated at the frequency of 2.45 GHz as $e_{r}$ = 3.9095 and σ = 0.339 S/m. Thermal properties provided in Table 1 for breast fat tissue were taken from [26].

### 2.2. FORA

**Figure 1:**
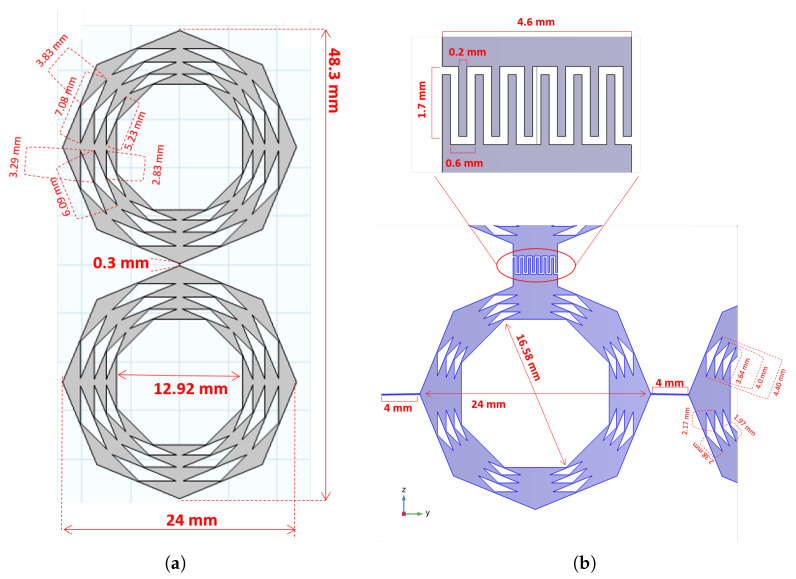
(**a**) FORA dipole element. (**b**) FORA—connected element and the inter-digital capacitor between the two elements in consecutive layers.

The antenna elements are based on fractal octagonal ring array geometry (referred to as FORA), a form of iterative octagon rings subdivided into sequences such that each sequence is a reduced size of the outer octagonal ring, with a scaling factor of 0.91. Two types of antennas were developed based on the proposed fractal elements, the dipole FORA antenna (Figure 1a) and the connected FORA antenna (Figure 1b). The dipole FORA antenna consists of two identical FORA elements matched to 50 Ω input impedance, such that the inter-element distance between the elements is 0.3 mm and the spacing between dipole arrays is one of the key parameters to balance between the room limitation and the mutual coupling. The dipole is fed through the 0.3 mm feed point between the FORA elements, see Figure 1a. The material of the antennas was selected as a perfectly electric conductor (PEC) throughout the simulations.

The connected FORA antenna is an array of FORA dipoles that are electrically connected, arranged such they are closely packed with an inter-element distance of 4 mm (<<λ/2 of the highest operating frequency-5 GHz-) between the arrays in one direction. The dimension of a single antenna element in the connected array should always be less than half the wavelength of the operating frequency. Furthermore, it is capacitively coupled in the longitudinal direction using inter-digital capacitors consisting of 6 fingers at each element end. The array in this form is no longer composed of separated resonant elements but can be considered to be a single antenna periodically fed and backed by a ground plane at a distance of ≈20 mm from the array elements [27]. The FORA—connected array is linearly polarized and matched to an input impedance of 180 Ω. The antennas were fed through the 4 mm feed points between the FORA elements, see Figure 1b.

**Figure 2:**
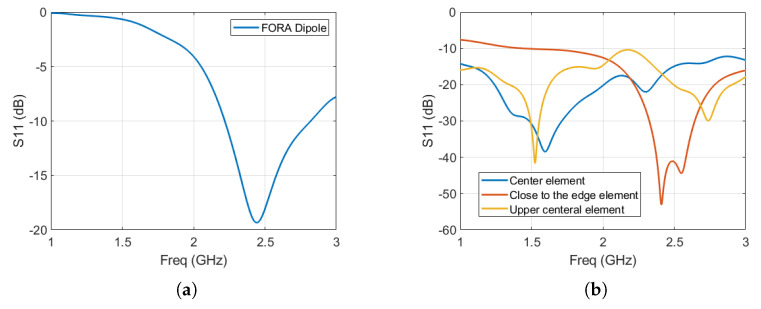
(**a**) Simulated S11 parameter as a function of frequency for a single FORA Dipole antenna in a linear array configuration, (**b**) and for different FORA—connected array elements.

The reflection coefficient performance of the two FORA antennas, namely the FORA dipole array and FORA—connected array, in the vicinity of the fat phantom are illustrated in Figure 2. As can be seen from both Figure 2a,b, the FORA dipole and the FORA—connected array are well matched with a return loss of less than −10 dB at the frequency of 2.45 GHz. This indicates that the incident power is efficiently transferred to the FORA antenna array and in turn, to the breast phantom. Figure 2b illustrates S11 parameters performance for different elements of the FORA—connected array fed at the center (the blue line), at distant from the center of the 3× 13 FORA connected array (yellow line) and the upper layer of the array just above the central element (orange line). Unlike the FORA—connected array where the S11 depends on the position of the elements in the array, the FORA dipole elements in the array exhibit similar S11 performance in the FORA dipole array deployed linearly, as can be seen in Figure 2a.

The FORA dipole element in a linear array consists of four elements and has a 3 dB beam width of 40.5${}^{\circ }$ in the elevation plane whereas 33.3${}^{\circ }$ in the azimuth plane. For the embedded element pattern in the connected array, there is a 3 dB beam width of 39.6${}^{\circ }$ in the elevation plane whereas 39.2${}^{\circ }$ in the azimuth plane. The maximum gains obtained for a single element at the center of the array are 1.7 dBi and 4.75 dBi for the dipole array and connected array, respectively. This is expected since the connected array has a ground plane and therefore has a higher gain that varies between 4 dBi to 8 dBi depending on the position of the element in the array. The bandwidths obtained by the FORA dipole and the connected array middle element are 24.5% and 81.6%, respectively. These bandwidths are enough for narrow-band hyperthermia applications.

### 2.3. HT Applicators

Different HT applicator designs were carefully investigated in this paper. First, a single layer of FORA dipoles was simulated, the results were analyzed, and the 2D, *z* = 0 plane was chosen for this scenario. Linear, circular, and cross arrays were implemented with FORA dipole antennas. Second, the 1-layer applicators which show the best performance were duplicated on the second layer and positioned such that they were symmetrical with respect to the *z* = 0 slice. The 2D evaluation was conducted on the same *z* = 0 plane, and the effect of the layer separation, dz, was investigated for the 2-layer dipole arrays. Finally, the connected FORA array was investigated for multiple layers and the number of antennas. The inter-antenna distances dy and dz are provided as multiples of the free space wavelength, $\lambda_{0}\approx$ 122.45 mm.

#### 2.3.1. Linear Array

**Figure 3:**
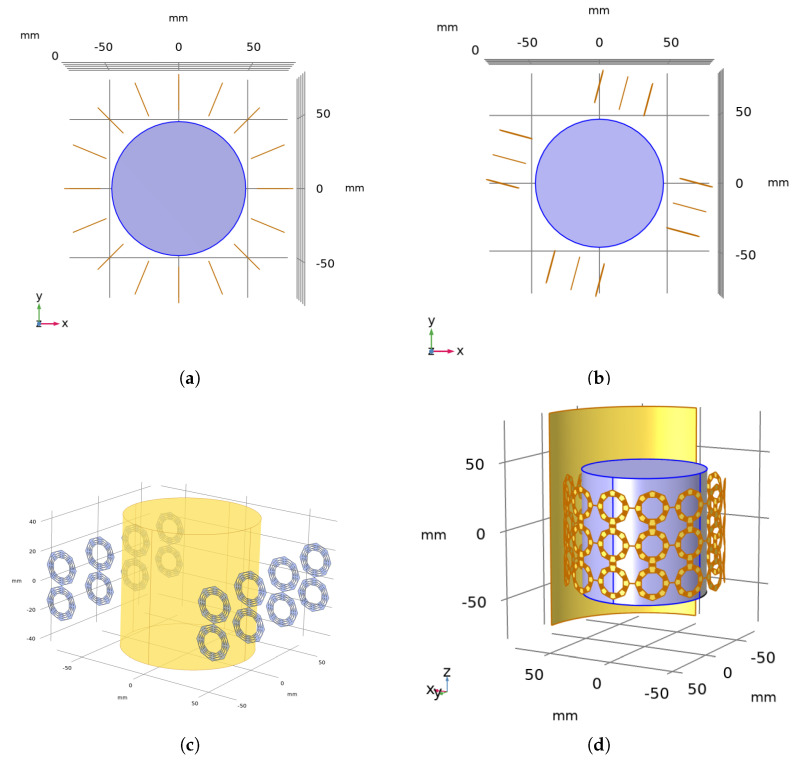
(**a**) Circular array with 16 FORA dipoles (top view). (**b**) Cross—array with 12 FORA dipoles (top view). (**c**) Linear array with eight FORA dipoles (side view). (**d**) Connected array with 39 FORA elements and three layers (side view).

The dipole antennas were placed on two opposite sides of the phantom with the same alignment. The distance between the first and the last antenna is dy, and the distance between the consecutive antennas is $\frac{dy}{N/2-1}$. A linear HT applicator with 8 antennas together with a cylindrical phantom can be seen in Figure 3c. An N—antenna linear array will be referred to as $LA^{N}$ for convenience. The analyzed parameters for the linear array are as follows:

- Number of antennas (N): 6, 8, 12, 16, 20 antennas
- dx: Distance between the tip of the antennas and the phantom
- dy: Distance between the first and the last antennas

#### 2.3.2. Circular Array

The dipole antennas were arranged in a circle of radius $r_{antenna}=(45$ mm +Δr) around the phantom ($r_{phantom}=45$ mm), and have an angular separation of 360/N degrees. A circular HT applicator with 8 antennas around a cylindrical phantom is shown in Figure 3a. An N—antenna circular array will be referred to as $CA^{N}$ for convenience. The analyzed parameters for the circular array are as follows:

- Number of antennas (N): 6, 8, 12, 16, 20 antennas
- Δr: Distance between the tip of the antennas and the phantom

#### 2.3.3. Cross—array

The dipole antennas were situated at the edges of a square centered on the axis of the phantom. The distance between the first and the last antenna is dy, and the distance between the consecutive antennas is $\frac{dy}{N/4-1}$. A cross HT applicator with 16 antennas is depicted in Figure 3b. An N—antenna Cross—array will be referred to as $XA^{N}$ for convenience. The analyzed parameters for the Cross—array are as follows:

- Number of antennas (N): 8, 12, 16, 20 antennas
- dx: Distance between the tip of the antennas and the phantom
- dy: Distance between the first and the last antennas

#### 2.3.4. Connected Array

The FORA elements are capacitively coupled via inter-digital capacitors in the longitudinal direction, and connected linearly through lumped ports laterally, as seen in Figure 1b and Figure 3d. A connected array with N antennas will be referred to as $ConA^{N}$ for simplicity. A total of 11 and 13 elements were analyzed for one layer, and the radii of connected arrays were 4 mm and 12.9 mm for the 11- and 13-element arrays, respectively. The curvature of the elements follows the given cylindrical geometry. Two and three layers for the 11-element and two, three, and five layers for 13-element arrays were investigated, namely 2 × 11, 2 × 13, 3 × 11 and 3 × 13 finite connected arrays with a backing reflector. The inter-element distances were kept the same and less than $0.5\lambda_{0}$ of the highest operational frequency (3 GHz). The side element and the middle element have one and two inter-digital capacitor arms in the longitudinal direction, respectively. The 2-layer ConA has two layers of side elements, while the 3-layer ConA has an additional layer of middle elements while the 5-layer ConA has three additional layers of middle elements. Furthermore, an 80 mm long cylindrical ground plane is situated at *r* = 80 mm to direct the radiation towards the phantom.

#### 2.3.5. Antenna Positions of FORA Dipole Arrays

In this paper, we analyze and compare the behavior of three different FORA dipole applicator designs. First, the effect of the FORA dipole orientation was investigated when the dipole is placed parallel and perpendicular to the phantom. Owing to the dipole symmetry, parallel and perpendicular orientations gave the same radiation pattern in free space and a similar pattern within the vicinity of the phantom. We adopt the perpendicular orientation of the FORA dipole for the rest of the paper since the inter-antenna distance can be adjusted much smaller with this configuration.

Second, each applicator design was optimized to give the best performance. To facilitate design comparison, the target centers were set on the positive x-axis, between (0, 0, 0) and (39, 0, 0) mm with increments of (1, 0, 0) mm. Keeping the target locations fixed, each applicator was axially rotated around the origin [11] and the TBR was analyzed. For the circular and the linear applicators, the positions shown in Figure 3a,c gave the individual best performance. For the cross applicator, it was observed that the best results for these targets were reached when the applicator was −15° rotated around the origin, as shown in Figure 3b, and the Cross—array results were taken from the geometry shown in Figure 3b.

### 2.4. Simulation Environment

The EM simulations and numerical calculations were implemented on Windows 10 Pro operating system with an Intel i7 processor and CPU 7800X with 3.50 GHz and 128 GB RAM. EM simulations were conducted using the finite element method (FEM) multiphysics solver COMSOL Multiphysics v6.0. *Electromagnetic Waves, Frequency Domain* physics inside *Radio Frequency* module was run on a single frequency in a frequency domain [28].

#### 2.4.1. Data Generation

The total electric field vector inside the breast phantom with N antenna excitations can be written as [29]:

(3) $$\begin{array}{c} {\vec{E}}_{tot}\left(r\right)=\sum_{i}^{N}a_{i}{\vec{E}}_{i}\left(r\right)e^{j\phi_{i}} \end{array}$$

where $\vec{E_{i}}\left(r\right)$ is the electric field vector inside the breast when only *i*th antenna is excited with unitary excitation and $a_{i}e^{j\phi_{i}}$ is the *i*th excitation coefficient with $\phi_{i}$ phase difference and $a_{i}$ amplitude. For every simulation case, N individual $\vec{E_{i}}\left(r\right)$ fields were exported for each antenna from the EM solver and sent to MATLAB. $\phi_{i}$ and $a_{i}$ are the parameters to be optimized for the desired focal point.

#### 2.4.2. SAR Optimization Metrics

In this section, the evaluation metrics to be used for the comparison are explained. Since the principal aim of this work was to focus the SAR on the desired target region, the metrics were devised based on SAR distributions. First, the 2D average spatial SAR operator is defined as follows:

(4) $$\begin{array}{cc} avSAR_{\Omega }=\frac{\sum_{\Omega }SAR}{\mathrm{Area}\;\mathrm{of}\Omega } & \;{W/\mathrm{kg}/m}^{2} \end{array}$$

where Ω is the surface of the 2D target region and av represents the averaged SAR over Ω. The main objective of the HT is to increase the SAR intensity at the target, while the healthy tissue SAR is kept to a minimum. To this end, target-to-breast ratio (TBR) and hotspot-to-target quotient (HTQ) [30] metrics were defined as follows:

(5) $$\begin{array}{cc} TBR & =\frac{avSAR_{target}}{avSAR_{breast}} \end{array}$$

(6) $$\begin{array}{cc} HTQ & =\frac{avSAR_{hotspot}}{avSAR_{target}} \end{array}$$

Defining the resolution as the width of the contour where the SAR intensity falls to half its maximum value, the resolution of the HT system inside the homogeneous fat cylinder phantom was ≈24 mm at 2.45 GHz. The desired target dimension is processed as half of this value: a square of 12 mm × 12 mm. The rest of the phantom region is divided into square grids with 12 mm side and $avSAR_{grid}$ is calculated for each of the grids. The hotspot region is chosen automatically as the grid with the highest $avSAR_{grid}$.

As mentioned before, PSO is prone to converge on a local best value depending on the specified initial conditions, which were random in our study. To obtain the underlying trend for each applicator case and to overcome the local convergence issue, a moving maximum filter with four consecutive points was applied to the obtained TBR values, and then a 6th degree polynomial fitting was applied.

#### 2.4.3. Particle Swarm Optimization

**Figure 4:**
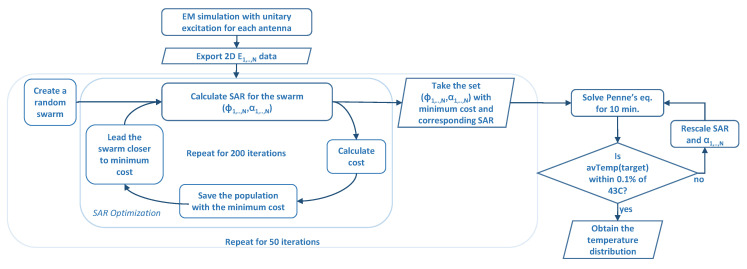
Flowchart of the optimization scheme.

Particle Swarm Optimization was used to optimize the antenna excitation parameters, i.e., the phase and the power of the individual antenna feed. PSO has previously been used as an optimization technique in HT [11,16,18,19]. It is a fast optimization technique, and it enables multi-parameter optimization and multi-objective cost functions. In particular, PSO with an inertia weight was used in this study [31]. It has been known that the random initialization of the parameters can cause local solutions, producing a different optimized parameter set every time PSO is applied. To overcome the sensitivity of the system to this issue, each PSO was conducted for 50 repetitions. For each repetition, a swarm size of 100 was iterated 200 times. The optimization process consists of multiple steps, and the corresponding flowchart is shown in Figure 4.

Multi-parameter optimization was implemented: phase difference and amplitude values were optimized together. Initially, the range of values that amplitudes can take was chosen as [0, 1] (V), and the range of values that phase differences can take as [−π, π] (rad). Cost function to be minimized was chosen as HTQ/TBR. From 50 repetitions, the optimized set of parameters giving the minimum cost was determined to be the solution. Using the optimized SAR distribution, Equation (1) was solved for ten minutes, and the corresponding temperature was calculated. With a feedback algorithm, SAR amplitude values were scaled and Equation (1) was calculated until the temperature level at the desired location increased to 43 °C in ten minutes. With this feedback algorithm, the final optimized amplitudes were recorded for the desired temperature levels.

#### 2.4.4. Temperature Calculation

Pennes’ Bio-heat Equation (1) was implemented using a finite difference time domain (FDTD) method in MATLAB. The resulting SAR distribution was fed into this equation to obtain the temperature change as a function of time. The time steps were two seconds and spanned over ten minutes, resulting in 300 time steps. The initial temperature of the phantom was assumed to be 37 °C. Spatial increments in both directions were taken as 1 mm. The Dirichlet boundary condition was used, where the boundary temperatures were equated to 37 °C. Using a feedback loop, the SAR was scaled until the temperature at the desired target reached 43 °C in 10 min. Then, this scaling was used to adjust the antenna excitation amplitudes. The amplitudes were converted into the antenna input power by $P_{i}\left[W\right]=a_{i}^{2}\left[V\right]/Z\left[\Omega \right]$, where $a_{i}$ was the excitation amplitude of the *i*th antenna and *Z* was the port impedance; *Z* = 50 Ω for the dipole and *Z* = 180 Ω for the connected array. The sum of the input powers can be written as $P_{tot}=\sum_{i}P_{i}$ [W].

## 3. Results and Discussion

The results are given first for 1-layer FORA dipole arrays. The best-performing 1-layer HT applicator designs are, then, duplicated to a second layer, and 2-layer FORA dipole array results are provided in Section 3.2. The results from connected FORA arrays are provided in Section 3.3.

**Figure 5:**
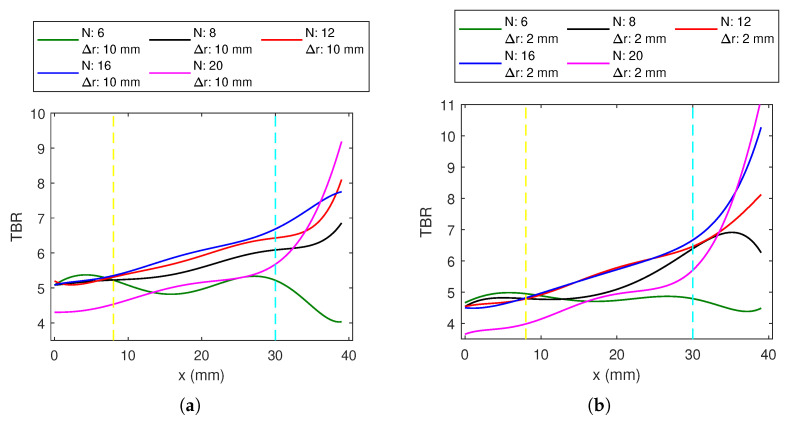
The TBR values evaluated at the target locations on the *x*-axis for 1-layer *N*-antenna circular dipole array (CA) with: (**a**) Δr = 10 mm, (**b**) Δr = 2 mm.

**Figure 6:**
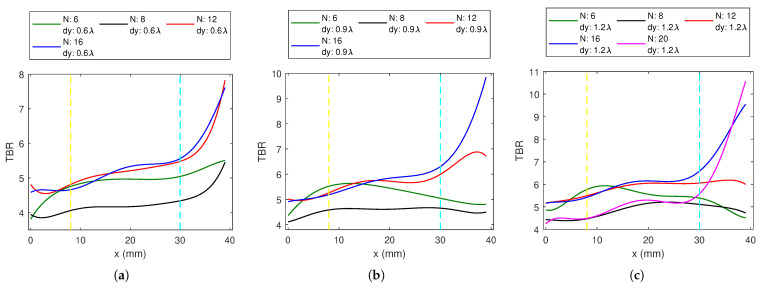
The TBR values evaluated at the target locations on the *x*-axis for 1-layer *N*-antenna linear dipole array (LA) with dx = 2 mm and (**a**) dy = 0.6 $\lambda_{0}$, (**b**) dy = 0.9 $\lambda_{0}$, (**c**) dy = 1.2 $\lambda_{0}$.

**Figure 7:**
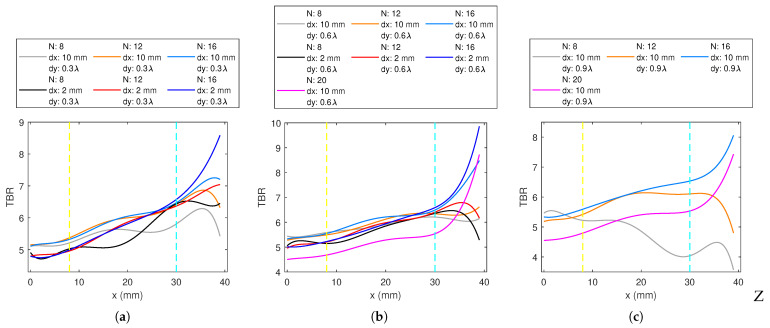
The TBR values evaluated at the target locations on the *x*-axis for 1-layer *N*-antenna cross-dipole array (XA) with: (**a**) dy = 0.3 $\lambda_{0}$, (**b**) dy = 0.6 $\lambda_{0}$, (**c**) dy = 0.9 $\lambda_{0}$.

**Figure 8:**
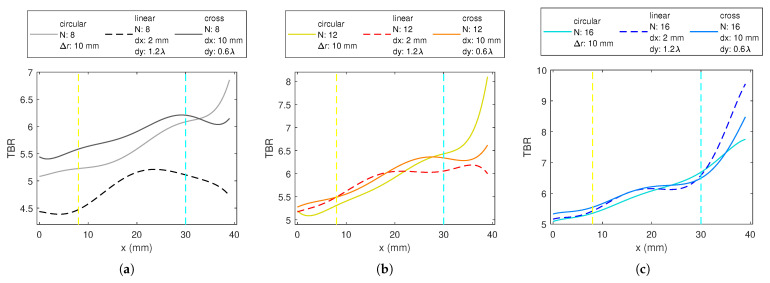
The TBR values evaluated at the target locations on the *x*-axis for: (**a**) 1-layer 8-antenna, (**b**) 1-layer 12-antenna, (**c**) 1-layer 16-antenna circular dipole array (CA), linear dipole array (LA) and cross-dipole array (XA) applicators.

**Figure 9:**
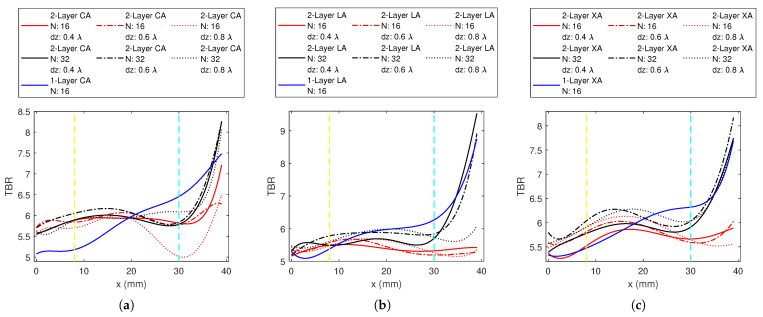
The TBR values evaluated at the target locations on the *x*-axis for: (**a**) 2-layer circular dipole array (CA) with Δr = 10 mm and *N* = 16, *N* = 32 and 1-layer 16-antenna CA with Δr = 10 mm, (**b**) 2-layer linear dipole array (LA) with dx = 2 mm and dy = 1.2 $\lambda_{0}$ and *N* = 16, *N* = 32 and 1-layer 16-antenna LA with dx = 2 mm and dy = 1.2 $\lambda_{0}$, and (**c**) 2-layer cross-dipole array (XA) with dx = 10 mm and dy = 0.6 $\lambda_{0}$ and *N* = 16, *N* = 32 and 1-layer 16-antenna XA with dx = 10 mm and dy = 0.6 $\lambda_{0}$.

**Figure 10:**
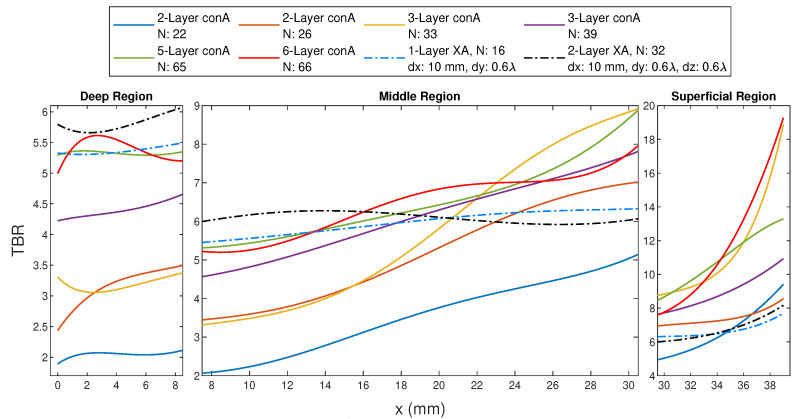
The TBR values evaluated at the target locations on the *x*-axis for FORA—connected array (ConA) with multi-layer, 1-layer 16-antenna cross-dipole array (XA) with dx = 10 mm and dy = 0.6 $\lambda_{0}$ and 2-layer 32-antenna cross-dipole array (XA) with dx = 10 mm, dy = 0.6 $\lambda_{0}$ and dz = 0.6 $\lambda_{0}$.

A common trend from the resulting data indicates a change in behavior of TBR with position, approximately at *x* = 8 mm and *x* = 30 mm. These are indicated with yellow and cyan lines in Figure 5, Figure 6, Figure 7, Figure 8, Figure 9 and Figure 10. We will refer to targets occurring in the region 0<x<8 mm as deep-seated, those occurring within 8<x<30 mm as the middle region, and those lying beyond *x* = 30 mm as the superficial region.

### 3.1. Results of 1-Layer FORA Dipole Arrays

The results of 1-layer circular, linear, and Cross—array HT applicators are presented in this section. The results for each applicator design are presented in separate sub-sections, then the results for N—antenna arrays and the best-performing applicator configuration are grouped and compared.

#### 3.1.1. Circular Applicator

Plots of TBR variation with the target position graphs are shown in Figure 5 for the 1-layer circular array. Figure 5a,b are given for Δr of 10 and 2 mm, respectively. The variation with respect to the number of antennas can be observed. Up to *N* = 16, the higher the number of antennas, the higher TBR values are obtained. $CA^{6}$ has higher TBR for deep-seated targets, and lower TBR for middle and superficial regions. Compared with other antenna numbers, although $CA^{6}$ has comparable TBR for deep-seated targets, it gives the lowest value for the remaining target locations. For Δr = 2 mm, $CA^{12}$ and $CA^{16}$ have almost the same values for the deep and the middle-region targets, while $CA^{16}$ provides higher TBR with the superficial regions. $CA^{20}$ has the lowest deep region performance among the investigated antenna numbers for the circular array and has high performance for the superficial regions.

CA with dx = 10 mm have higher TBR at deep and middle regions; however, the performance of CA with dx = 2 mm is better in superficial regions.

#### 3.1.2. Linear Applicator

For the 1-layer linear array, dx = 10 mm resulted in smaller TBR than for dx = 2 mm in all cases, and therefore the associated results are omitted. For a fixed dx = 2 mm, TBR plots for different dy values with changing antenna numbers are shown in Figure 6. In contrast with the circular array, the 8-antenna linear array has lower TBR than the 6-antenna array over the phantom for three dy values. $LA^{12}$ and $LA^{16}$ behave similarly for deep and middle targets, while $LA^{16}$ has higher TBR for superficial regions. It can be observed that, in general, as dy increases, TBR also increases. *N* = 20 was evaluated only for dx = 2 mm and dy = 1.2 $\lambda_{0}$ (Figure 6c). $LA^{20}$ has poor deep and middle-region performance but TBR values drastically increase for superficial regions.

#### 3.1.3. Cross Applicator

Figure 7 shows the TBR vs. target location plots of constant dy cases. $XA^{16}$TBR values are predominantly superior in all the cases, followed by $XA^{12}$. The slope of $XA^{16}$ increases while the targets become closer to the surface, $XA^{12}$ and $XA^{16}$ follow a very similar trend in the deep and middle regions.

For $XA^{8}$, dx = 10 mm alignment provided higher TBR than dx = 2 mm in the deep and middle regions. For the same dy values, however, dx = 2 mm shows better performance for the superficial regions. dy = 0.3 $\lambda_{0}$ exhibits lower TBR than dy = 0.6 $\lambda_{0}$ in the deep and middle regions. For both $XA^{8}$ and $XA^{12}$, although comparable to others in deep-seated regions, dx = 10 mm and dy = 0.9 $\lambda_{0}$ provides much lower TBR in the superficial region (Figure 7c). Up to the superficial region, for $XA^{12}$, dx = 10 mm was superior to dx = 2 mm, and dy = 0.6 $\lambda_{0}$ was superior to dy = 0.3 $\lambda_{0}$ (Figure 7a,b). In the superficial region, the performance of dx = 2 mm is superior. All the cases of $XA^{16}$ follow a similar trend. In the deep and middle regions, the combination dx = 10 mm and dy = 0.6 $\lambda_{0}$ results in a higher TBR, and dy = 0.9 $\lambda_{0}$ was close to dy = 0.6 $\lambda_{0}$, contrary to the other antenna numbers. In the superficial regions, the TBR performance with dx = 2 mm increases.

$XA^{20}$ results are given for dx = 10 mm case for both dy = 0.6 $\lambda_{0}$ and 0.9 $\lambda_{0}$ designs. $XA^{20}$ performance was low in the deep and middle regions and above *N* = 8 and 12 applicators in the superficial regions (Figure 7b,c).

The cases with the highest TBR values for different applicator designs are plotted in Figure 8 for *N* = 8, 12, and 16. Cross and circular 1-layer applicators with the same *N*, provide, in general, similar trends of TBR, while the values for circular arrays are slightly inferior to those from cross arrays for deep and middle-region targets, and for *N* = 8 and 12, much higher in the superficial regions (Figure 8a,c). The linear applicator does not perform as well as the others when *N* = 8 (Figure 8a). The performance of the $LA^{12}$ configuration is comparable to that of $XA^{12}$ in the inner half of the phantom but inferior to the other configurations in the outer half (Figure 8b). For 16 antennas, its performance is better than the others for superficial regions (Figure 8c). For deep and middle regions with *N* = 16, there is no distinct difference between the circular, cross, and linear applicator structures in terms of TBR.

**Figure 11:**
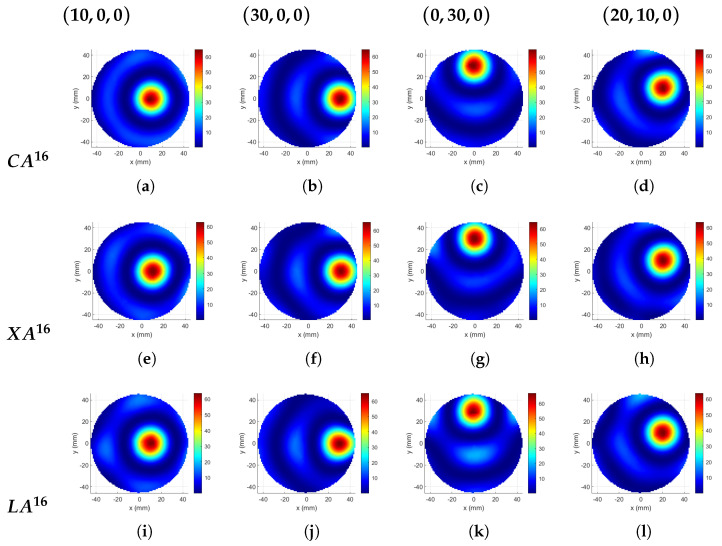
SAR distributions [W/kg] focused at (10, 0, 0), (30, 0, 0), (0, 30, 0), and (20, 10, 0) mm positions, and obtained with 1-layer of FORA dipole elements. (**a**–**d**) Circular array with 16 antennas and Δr = 10 mm. (**e**–**h**) Cross—array with 16 antennas, dx = 10 mm, and dy = 0.6 $\lambda_{0}$. (**i**–**l**) Linear array with 16 antennas, dx = 2 mm, and dy = 1.2 $\lambda_{0}$.

The applicator designs given in Figure 8c were used to focus on four targets and the resulting SAR distributions are shown in Figure 11. The corresponding TBR and $P_{tot}$ values are given in Table 2. The first and the second targets in the table are on the x-axis, and they can be referred to as the deep and the superficial regions. TBR values are consistent with each other such that the linear array has the highest value and the circular array has the lowest value. The deep target TBR was lower than the superficial target values. Although the third target was at the same distance from the origin as the second target, the linear array result changed drastically. This is because the position of the linear array was assumed as stated previously in this paper such that the best performance occurs along the x-axis. Since the third target is rotated 90° rotated from the x-axis, it was expected that the TBR value of the linear array becomes lower. The position of the Cross—array was also arranged for the x-axis, but since it is symmetrical on 4 quarters, there was almost no change in the TBR value. The result of the circular array does not change for the third target due to circular symmetry. The fourth target was in the middle region and has an angle of 25.5° with the x-axis. Cross and linear arrays give higher TBR than the circular for the fourth target.

**Figure 12:**
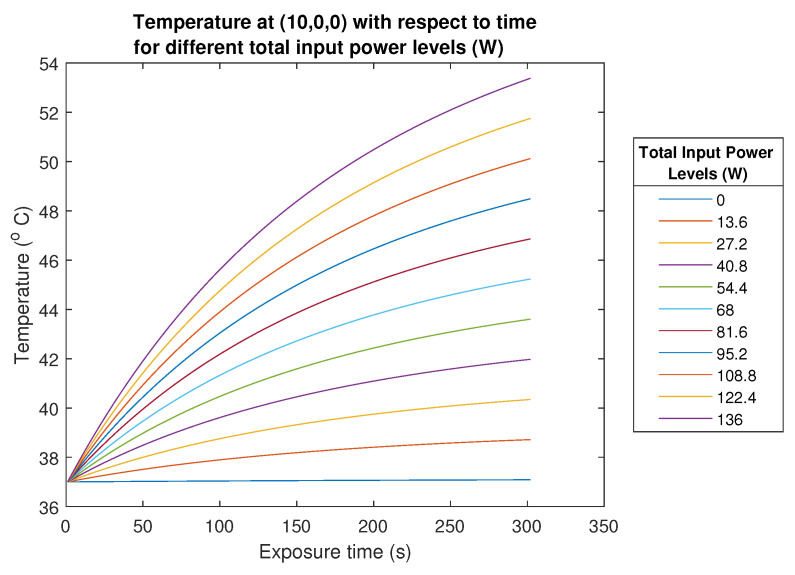
Temperature (℃) at (10, 0, 0) mm target point as a function of exposure time for different total input power levels (W).

The circular array has the lowest power requirement to reach 43 °C in 10 min and the linear array has the highest $P_{tot}$. Please note that power requirements were not optimized in this study, and these values were obtained with the procedure explained in Section 2.4.4. Concerning the 1-layer 16-antenna circular FORA dipole array applicator, with 68 W total input power, after ten minutes of SAR exposure, the temperature at the (10, 0, 0) target point increases to 45.2 ℃, and the average temperature at the target region (12 mm × 12 mm region centered at (10, 0, 0) point) becomes 43 ℃. The total input power, then, was scaled from 0 W to 136 W with 13.6 W increments and the temperature level at the target point was calculated for the corresponding scaled SAR distributions to show the temperature change for different power levels. The temperature at the target is shown in Figure 12 as a function of exposure time for each input power level (W) provided in the legend.

For 1-layer arrays, in general, a larger number of antennas gives higher TBR, which was an expected result due to increased optimization sensitivity, directivity, and gain. The number of antennas can be increased until the mutual coupling limits are reached. Arrays with 20 antennas exhibited inferior performance when compared to other applicators with a smaller number of antennas in the array.

### 3.2. Results of 2-Layer FORA Dipole Arrays

The best HT applicator designs obtained from the 1-layer application were: the 16-antenna circular array with Δr = 10 mm, 16-antenna Cross—array with dx = 10 mm and dy = 0.6 $\lambda_{0}$, and 16-antenna linear array with dx = 2 mm and dy = 1.2 $\lambda_{0}$ (also shown in Figure 8c). This suggests that among the 1-layer applicators, 16-antenna arrays perform the best. In this section, we duplicated these 1-layer antenna arrays to a second layer and set the inter-layer distance to 0.4 $\lambda_{0}$, 0.6 $\lambda_{0}$, and 0.8 $\lambda_{0}$, while maintaining the symmetry around the *z* = 0 plane. Furthermore, the same procedure was repeated by decreasing the number of antennas in each layer to half to understand whether *N* = 16 should be maintained as the total number of antennas or as the number of antennas in one layer. Therefore, 2-layer 32-antenna and 2-layer 16-antenna applicators were explored. Figure 9 shows the 2-layer results and the corresponding 1-layer best-case result.

Among the 2-layer CAs, the 32-antenna arrays show superior results to the array with 16 antennas as shown in Figure 9a. For N=16, dz = 0.6 $\lambda_{0}$ provides higher TBR than dz = 0.4 $\lambda_{0}$, while dz = 0.8 $\lambda_{0}$ gives the lowest TBR values. For *N* = 32, dz = 0.6 $\lambda_{0}$ provides higher TBR than both dz = 0.4 $\lambda_{0}$ and dz = 0.8 $\lambda_{0}$. 2-layer $CA^{32}$ with dz = 0.4 $\lambda_{0}$ has the highest TBR value among the explored designs. The 2-layer CA shows inferior results to the 1-layer best CA up to the middle region and performs better in the superficial region. At the most superficial target that was explored, the 2-layer $CA^{32}$ becomes comparable to the 1-layer $CA^{16}$.

Comparing 2-layer linear arrays given in Figure 9b, $LA^{32}$ was superior to $LA^{16}$. dz = 0.8 $\lambda_{0}$ has the lowest and dz = 0.4 $\lambda_{0}$ has the highest superficial performance. dz = 0.6 $\lambda_{0}$ has higher TBR for most of the remaining regions for $LA^{32}$. $LA^{32}$ has higher TBR values than the 1-layer $LA^{16}$ in the deep region, and comparable results at the most superficial target that was investigated. However, over most of the phantom, the 1-layer $LA^{16}$ has better performance.

In Figure 9c, the 32-antenna array with dz = 0.8 $\lambda_{0}$ has the highest TBR value, followed by dz = 0.6 $\lambda_{0}$. $XA^{32}$ was superior to its counterpart with 16 antennas that have the same dz distance. The 2-layer cross applicators investigated, except $XA^{16}$ with dz = 0.4 $\lambda_{0}$, show better performance than the 1-layer $XA^{16}$ in the deep region and the first half of the middle region, but the 1-layer $XA^{16}$ shows better performance in the outer half of the phantom. The 2-layer $XA^{32}$ catches up with the 1-layer $XA^{16}$ performance at the outermost target regions.

Table 3 provides the TBR values and $P_{tot}$ –the power requirement for the target to reach 43 °C in 10 min.– of 2-layer $CA^{32}$ with Δr = 10 mm and dz = 0.6 $\lambda_{0}$, and the 2-layer $XA^{32}$ with dx = 10 mm, dy = 0.6 $\lambda_{0}$, and dz = 0.8 $\lambda_{0}$ for targets (10, 0, 0) mm and (30, 0, 0) mm. In the deep target, although the TBR values were higher for 2-layer applicators than for the 1-layer, the increase in the $P_{tot}$ values was greater. At the superficial target, 2-layer applicators both exhibit lower performance and higher power demand. Please note that the power requirement was not optimized in this study.

Duplicating the 1-layer array with the best results onto the second layer provided better performance in the inner half of the phantom for circular and cross applicators than their 1-layer counterparts, and only in the deep region for the linear applicator. Although the 32-antenna 2-layer applicators reach and even exceed the performance of the 1-layer arrays in the outermost targets, their performance in the outer half of the phantom was inferior to 1-layer applicators. For a deep-seated target, it was better to use multilayers; however, 1-layer FORA dipole applicators with 16 antennas perform better for the remaining phantom regions.

### 3.3. Results of Multi-Layer FORA—Connected Arrays

The connected FORA array results are given in Figure 10. In this plot, TBR values are given separately for three regions, and the sub-plots of each region are scaled to better discern the results. The TBR value was higher for the higher number of layers in the connected array, although the performance of the 3- and 5-layer 13-antenna connected array becomes comparable around *x* = 20 mm. TBR values were higher for the 13-antenna ConA than the ones with 11 antennas for the same number of layers, except for the outermost targets. This was a similar situation to the dipole circular array, where Δr = 2 mm shows better performance over Δr = 10 mm in the outermost targets. The 5-layer 13-antenna ConA and 6-layer 11-antenna ConA are especially given together in Figure 10 since their number of antennas is close. The 65- and 66-antenna arrays show similar behavior in the inner half of the phantom. The TBR resulting from the 65-antenna array shows a more monotonic increase in the second half of the phantom, while the 66-antenna array shows better performance in the outermost targets. The investigated connected arrays show inferior results compared to the 1-layer $CA^{16}$ with Δr = 10 mm in the deep and the middle regions, except for $ConA^{65}$ and $ConA^{66}$, which resulted in comparable results. In the superficial region, the 3-, 5-, and 6-layer connected arrays show better performance than the dipole circular array.

In Figure 10, the 1- and 2-layer cross-dipole array results as well as the connected array results are shown together. In the deep region, 2-layer $XA^{16}$ is superior and followed by $conA^{66}$, $conA^{65}$ and 1-layer $XA^{16}$. In the middle region, the behavior of all the applicators changes. In the superficial region, $conA^{66}$ and $conA^{33}$ are superior and followed by $conA^{65}$, and the 1- and 2-layer cross-dipole array performances are inferior to most of the explored connected arrays. The $conA^{66}$ provides the overall better performance, suggesting the high number of antennas constituting the connected array demonstrate better focusing capability. Although the number of antennas within $conA^{65}$ is very close to 66 antennas, one can conclude that the higher number of layers of the connected array also demonstrates better performance. Also, when the distance between the antenna and the phantom is small, the focusing performance at the superficial regions increases as in $conA^{33}$ and $conA^{66}$. Adding higher layers than two was not possible for the given arrangement of the dipole array. Therefore, more layers could not be compared.

Table 3 provides the TBR and $P_{tot}$ values of $ConA^{39}$, $ConA^{65}$ and $ConA^{66}$ at a deep and superficial target. Comparing Table 2 and Table 3, 5- and 6-layer ConA have comparable deep target performance with the best-case 1-layer applicators, while $ConA^{39}$ was inferior. In superficial regions, three connected arrays show superior performance to the 1-layer dipole applicators. Power requirements of ConA were higher than 1-layer dipole applicators and comparable to 2-layer dipole applicators.

In beamforming studies, the inter-antenna distance is more important than the total area that the antennas span. In a medical application, however, there is limited available space, and the total area that the antenna span becomes an important issue. This is why, in this paper, instead of the inter-antenna distance, dy, the distance between the first and the last antennas, is used as one of the parameters. The effects on the applicator performance of the other parameters, such as the number of antennas, were compared for a fixed antenna space. The total applicator distance on the *x*-axis was 158 mm when dx = 10 mm and 142 mm when dx = 2 mm and the largest antenna separation that was investigated was dy = 1.2 λ≈ = 147 mm in the *y*-direction. Therefore, for the best-performing 1-layer dipole linear array, the area of the applicator was 147 mm × 142 mm, and it was 158 mm × 158 mm for the circular array. This paper shows a comparison between different FORA element arrays on a cylindrical fat phantom of diameter 90 mm. The results of best-performing HT applicators might be different for a bigger phantom or a realistic breast phantom.

### 3.4. Experimental Results

The purpose of the experiment was to increase the temperature of the phantom at (9, 21, 0) mm, where the center of the phantom was chosen as the origin. The fat-mimicking phantom has a radius of 45 mm and height of 90 mm and is cut into two equal pieces at z=0 slice. To mimic the fat tissue, 80% oil-in-gelatin mixture was prepared according to the instructions given in [32]. The dielectric properties of the manufactured phantom were measured with a DAK probe to have $\epsilon_{r}$ = 3.77 and σ = 0.035 S/m at 2.45 GHz. Three measurement points were chosen at z=0 slice as: (30, 0) mm, (25, 25) mm, (9, 21) mm. For each point, three more points were selected 90 degrees apart, symmetrical with respect to the origin. The thirteenth measurement point was selected at the origin.

**Figure 13:**
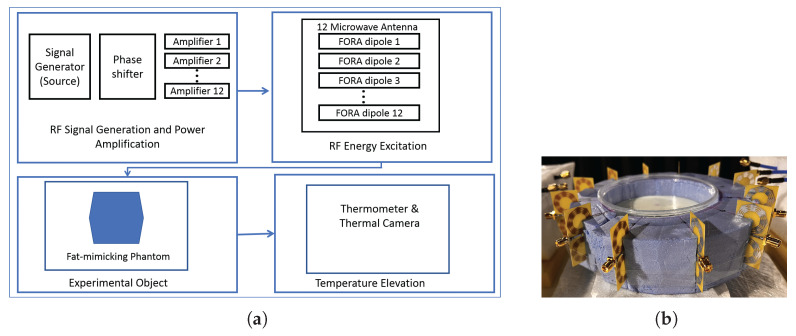
(**a**) The block diagram of the experimental system. (**b**) The circular FORA dipole array prototype with the bottom half of the fat-mimicking phantom in the middle.

The HT applicator was comprised of 12 FORA dipole antennas arranged in a circular array and enclosed in expanded polyethylene foam, as shown in Figure 13b. The block diagram of the components of the experimental system is given in Figure 13a. Each antenna was connected to an individual 10W RF power amplifier (HI Microwave Technology, China HIPA02034040) and the corresponding channels of the phase shifter (HI Microwave Technology, China HPS-1700T6000M, with 20 dB loss at each channel) providing a specific relative phase shift. Phase shifter was fed with 16 dBm signal with 2.45 GHz generated by the microwave source (Agilent Technologies, USA E8257D). The excitation parameters were further optimized according to the antenna maximum power inputs allowed by the RF system. Using a Vector Network Analyzer (Keysight, USA M9018A PXIe Chasis), each signal at the antenna input was fine-tuned to overcome any imbalance over each amplifier. Table 4 details the ideal magnitude and phase values required for each element in the array.

**Figure 14:**
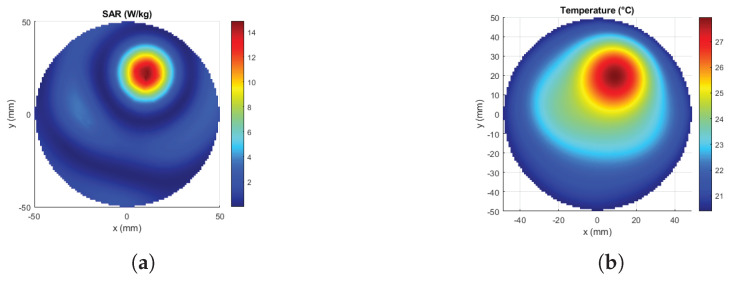
Computational results of the experimental setup. (**a**) Optimized SAR distribution (W/kg), (**b**) Temperature profile after 60 min (°C).

The maximum available antenna input power was 4 W due to the loss at the phase shifter, while 70 W was required in the simulation results to reach 43 °C from 37 °C in ten minutes. The experiment was run for 60 min when the phantom initial temperature was 18 °C and the room temperature was 20 °C. Computational results are also given for 60 min of treatment. The computed temperature distribution and the thermal camera (Guide ZC04C2001351) image are compared to verify the thermal effect generated by the HT applicator in a 2D plane (XY-plane). Figure 14 shows the optimized SAR distribution of the simulation for the experimental setup, and the associated temperature profile after an hour.

**Figure 15:**
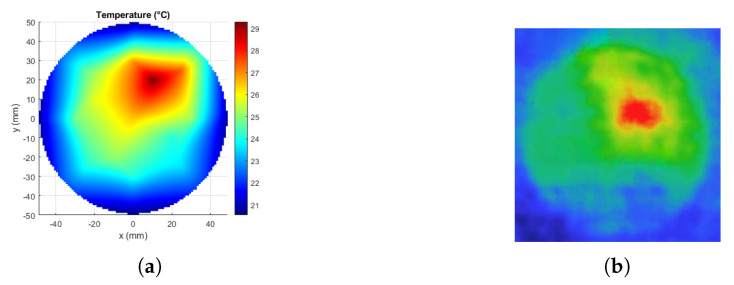
Experimental results. (**a**) Temperature distribution interpolated from the 13 points of the thermometer readings (°C), (**b**) Thermal camera image.

The phantom was taken outside of the applicator after 60 min, the top half of the phantom was put aside, and all the data were taken from the bottom piece at z=0 slice. First, the thermal image was taken and then, thermometer readings were recorded at 13 points, and these 13 values were interpolated in MATLAB to visualize the temperature distribution. Figure 15a shows the temperature profile. The temperature profile from the thermal camera is shown in Figure 15b.

The utilized thermal camera during the experiment did not provide specific temperature values or color bars. Therefore, the real temperature values could not be obtained. The highest temperature position, on the other hand, was conceivable and well matched with the computed results. To obtain the specific temperature values, thermometer readings were provided from 13 discrete locations and the values of these positions also match well with the expected result. Considering the two data acquisition techniques, both the real temperature values and the surface temperature distribution, the experimental results verify the computational results. The difference between the temperature distributions of the computed and the experimental results can be due to the inhomogeneity of the phantom and the unknown thermal parameters.

## 4. Conclusions

In this paper, we presented fractal octagonal (FORA) elements adopted for two types of antenna arrays, the sparse array and the connected array. The former is referred to as the FORA dipole array, and the latter is the FORA—connected array. The choice for FORA elements was due to their characteristics, which enable tailoring these elements for such types of arrays. This paper investigated FORA antenna elements as breast hyperthermia applicators for deep and superficial seated targets. The phantom was modeled as a fatty tissue as it provides a homogeneous environment for such a comparative analysis.

FORA elements were used both as 1- and 2-layer dipole arrays and multi-layer connected arrays. First, the 1-layer dipole antenna arrays were analyzed, and the best-performing designs were shown. FORA dipole antenna performance aligned in a linear, cross, and circular array were examined compared to each other’s performance to selectively minimize hot spots while focusing the energy on a particular target in the phantom under quest. One-layer 16-antenna dipole arrays were found to be superior to the other one-layer antenna arrays with lower or higher numbers of antennas for all dipole array configurations. Duplicating these best cases showed that the 2-layer dipole array performs better in the deep-seated regions. Two-layer circular and cross arrays performed superior to the two-layer linear array in the deep-seated regions. Based on these results, 1- and 2-layer cross arrays were compared to connected arrays. Then, multi-layer connected FORA arrays were investigated for 2-,3-,5- and 6-layers. It was found that their results were superior to the 1-layer dipole array in the superficial region of the phantom. In terms of target-to-breast SAR ratio TBR performance, one can conclude that the 2-layer dipole FORA array would be a better choice for reaching deep-tissue targets, while the multi-layer connected arrays should be chosen for the superficial regions. However, the power requirements of these multi-layer connected arrays were higher than the examined 1-layer HT dipole applicators. The experimental results verify the use of FORA antenna in microwave hyperthermia application.

## Figures and Tables

**Table 1:** The Debye parameters of the fat tissue dielectric properties. Thermal properties of the fat tissue.

**Debye Model**	$\epsilon_{\infty }$	Δϵ	$\sigma_{s}$ (S/m)	τ (ps)
Parameters	2.85	1.10	0.01	13.00
**Dielectric**	$\epsilon_{r}$	σ (S/m)		
**Properties at 2.45 GHz**	3.9095	0.339		
**Thermal**	ρ (${\mathrm{kg}/m}^{3}$)	$C_{p}$ (J/kg/K)	k (W/m/K)	Qm (${W/m}^{3}$)
**Properties**	932	2220	0.17	458

**Table 2:** The TBR and total antenna input power ($P_{tot}$) results of the best-performing 1-layer FORA dipole applicators focused on different target locations.

	Target Position (mm)	(10, 0, 0)	(30, 0, 0)	(0, 30, 0)	(20, 10, 0)
Applicator					
		TBR			
$CA^{16}$, Δr = 10 mm		5.26	6.45	6.45	5.54
$XA^{16}$, dx = 10 mm and dy = 0.6 $\lambda_{0}$		5.56	6.32	6.34	5.80
$LA^{16}$, dx = 2 mm and dy = 1.2 $\lambda_{0}$		5.59	6.58	5.76	5.81
		$P_{\mathrm{tot}}\left[W\right]$			
$CA^{16}$, Δr = 10 mm		68	63	78	89
$XA^{16}$, dx = 10 mm and dy = 0.6 $\lambda_{0}$		69	109	94	158
$LA^{16}$, dx = 2 mm and dy = 1.2 $\lambda_{0}$		162	174	187	219

**Table 3:** The TBR and total antenna power $P_{tot}$ results of the best-performing 2-layer FORA dipole applicators and FORA—connected array applicators focused on different target locations.

	Target Position (mm)	(10, 0, 0)	(30, 0, 0)
Applicator			
		TBR	
2-layer $CA^{32}$, Δr = 10 mm, dz = 0.6 $\lambda_{0}$		6.10	5.84
2-layer $XA^{32}$, dx = 10 mm, dy = 0.6 $\lambda_{0}$, dz = 0.8 $\lambda_{0}$		6.02	6.04
3-layer $ConA^{33}$, Δr = 4 mm		4.82	7.72
5-layer $ConA^{65}$, Δr = 12.9 mm		5.44	8.67
6-layer $ConA^{66}$, Δr = 4 mm		5.26	7.76
		$P_{\mathrm{tot}}$ **[W]**	
2-layer $CA^{32}$, Δr = 10 mm, dz = 0.6 $\lambda_{0}$		270	150
2-layer $XA^{32}$, dx = 10 mm, dy = 0.6 $\lambda_{0}$, dz = 0.8 $\lambda_{0}$		271	554
3-layer $ConA^{33}$, Δr = 4 mm		197	206
5-layer $ConA^{65}$, Δr = 12.9 mm		332	319
6-layer $ConA^{66}$, Δr = 4 mm		176	189

**Table 4:** Optimized antenna excitations values for the experiment.

Antenna Number												
	1	2	3	4	5	6	7	8	9	10	11	12
Phase (°)	21	132	65	70	42	−103	−130	−90	−138	−60	35	114
Power (W)	0.80	1.61	1.02	1.86	2.27	1.67	3.32	3.73	2.88	0.72	3.09	1.58

## Data Availability

Not applicable.

## Abbreviations

The following abbreviations are used in this manuscript:

HT	Hyperthermia
SAR	Specific Absorption Rate
FORA	Fractal octagonal ring array
EM	Electromagnetic
TR	Time Reversal
iw-PSO	inertia weighted-Particle Swarm Optimization
HTP	Hyperthermia Treatment Planning
FDTD	Finite Difference Time Domain
FEM	Finite Element Method
ISM	Industrial Scientific Medical
MRI	Magnetic Resonance Imaging
TBR	Target-to-breast ratio
HTQ	Hotspot-to-target quotient
CA	Circular Array
LA	Linear Array
XA	Cross—array
ConA	Connected Array

## References

[1] van der Zee J., González D., van Rhoon G.C., van Dijk J.D., van Putten W.L., Hart A.A. (2000). Comparison of radiotherapy alone with radiotherapy plus hyperthermia in locally advanced pelvic tumours: A prospective, randomised, multicentre trial. Lancet.

[2] Jha S., Sharma P.K., Malviya R. (2016). Hyperthermia: Role and risk factor for cancer treatment. Achiev. Life Sci..

[3] Alexander H. (2001). Isolation perfusion. Cancer Princ. Pract. Oncol..

[4] Lee A.H. (2005). Why is carcinoma of the breast more frequent in the upper outer quadrant? A case series based on needle core biopsy diagnoses. Breast.

[5] Iero D.A., Isernia T., Morabito A.F., Catapano I., Crocco L. (2010). Optimal constrained field focusing for hyperthermia cancer therapy: A feasibility assessment on realistic phantoms. Prog. Electromagn. Res..

[6] Isernia T., Di Iorio P., Soldovieri F. (2000). An effective approach for the optimal focusing of array fields subject to arbitrary upper bounds. IEEE Trans. Antennas Propag..

[7] Iero D.A., Crocco L., Isernia T. (2013). Thermal and microwave constrained focusing for patient-specific breast cancer hyperthermia: A robustness assessment. IEEE Trans. Antennas Propag..

[8] Yildiz G., Yasar H., Uslu I.E., Demirel Y., Akinci M.N., Yilmaz T., Akduman I. (2022). Antenna Excitation Optimization with Deep Learning for Microwave Breast Cancer Hyperthermia. Sensors.

[9] Fenn A.J. (1999). An adaptive microwave phased array for targeted heating of deep tumours in intact breast: Animal study results. Int. J. Hyperth..

[10] Altintas G., Akduman I., Janjic A., Yilmaz T. (2021). A Novel Approach on Microwave Hyperthermia. Diagnostics.

[11] Yildiz G., Yilmaz T., Akduman I. (2022). Rotationally Adjustable Hyperthermia Applicators: A Computational Comparative Study of Circular and Linear Array Applicators. Diagnostics.

[12] Curto S., Garcia-Miquel A., Suh M., Vidal N., Lopez-Villegas J.M., Prakash P. (2017). Design and characterisation of a phased antenna array for intact breast hyperthermia. Int. J. Hyperth..

[13] Xu L., Wang X. (2019). Focused microwave breast hyperthermia monitored by thermoacoustic imaging: A computational feasibility study applying realistic breast phantoms. IEEE J. Electromagn. Microwaves Med. Biol..

[14] Li J., Xu L., Wang X. (2019). A computational study on number of elements in antenna array for focused microwave breast hyperthermia. Proceedings of the 2019 IEEE MTT-S International Microwave Biomedical Conference (IMBioC), Nanjing, China, 6–8 May 2019.

[15] Li J., Wang B., Zhang D., Li C., Zhu Y., Zou Y., Chen B., Wu T., Wang X. (2021). A preclinical system prototype for focused microwave breast hyperthermia guided by compressive thermoacoustic tomography. IEEE Trans. Biomed. Eng..

[16] Nguyen P.T., Abbosh A., Crozier S. (2017). Three-Dimensional Microwave Hyperthermia for Breast Cancer Treatment in a Realistic Environment Using Particle Swarm Optimization. IEEE Trans. Biomed. Eng..

[17] Elkayal H.A., Ismail N.E. (2021). Efficient focusing of microwave hyperthermia for small deep-seated breast tumors treatment using particle swarm optimization. Comput. Methods Biomech. Biomed. Eng..

[18] Nguyen P.T., Abbosh A.M., Crozier S. (2017). 3-D Focused Microwave Hyperthermia for Breast Cancer Treatment With Experimental Validation. IEEE Trans. Antennas Propag..

[19] Nguyen P.T., Abbosh A., Crozier S. (2015). Microwave Hyperthermia for Breast Cancer Treatment Using Electromagnetic and Thermal Focusing Tested on Realistic Breast Models and Antenna Arrays. IEEE Trans. Antennas Propag..

[20] Farhat E.O., Adami K.Z., Zhang Y., Brown A.K., Sammut C.V. (2013). Ultra-wideband tightly coupled fractal octagonal phased array antenna. Proceedings of the 2013 International Conference on Electromagnetics in Advanced Applications (ICEAA), Turin, Italy, 9–13 September 2013.

[21] Farhat I.O., Adami K.Z., Sammut C.V. (2015). Near-Field to Far-Field pattern measurements for a mid-aperture FR-ORA array. Proceedings of the 2015 IEEE Conference on Antenna Measurements & Applications (CAMA), Chiang Mai, Thailand, 30 November–2 December 2015.

[22] Wheeler H. (1965). Simple relations derived fom a phased-array antenna made of an infinite current sheet. IEEE Trans. Antennas Propag..

[23] Pennes H.H. (1998). Analysis of Tissue and Arterial Blood Temperatures in the Resting Human Forearm. J. Appl. Physiol..

[24] Zastrow E., Davis S., Lazebnik M., Kelcz F., Van Veen B., Hagness S. (2008). Development of Anatomically Realistic Numerical Breast Phantoms With Accurate Dielectric Properties for Modeling Microwave Interactions With the Human Breast. IEEE Trans. Biomed. Eng..

[25] (2008). Phantom Repository. https://uwcem.ece.wisc.edu/phantomRepository.html.

[26] Said Camilleri J., Farrugia L., Curto S., Rodrigues D.B., Farina L., Caruana Dingli G., Bonello J., Farhat I., Sammut C.V. (2022). Review of Thermal and Physiological Properties of Human Breast Tissue. Sensors.

[27] Farhat I., Cutajar D., Adami K.Z., Sammut C., Abela J. (2018). Characterization of 36 meter square mid-frequency radio astronomy prototype antenna array. Proceedings of the 2018 IEEE Conference on Antenna Measurements & Applications (CAMA), Vasteras, Sweden, 3–6 September 2018.

[28] COMSOL RF Module User’s Guide. https://doc.comsol.com/5.4/doc/com.comsol.help.rf/RFModuleUsersGuide.pdf.

[29] Canters R., Wust P., Bakker J., Van Rhoon G. (2009). A literature survey on indicators for characterisation and optimisation of SAR distributions in deep hyperthermia, a plea for standardisation. Int. J. Hyperth..

[30] Sumser K., Bellizzi G.G., van Rhoon G.C., Paulides M.M. (2020). The Potential of Adjusting Water Bolus Liquid Properties for Economic and Precise MR Thermometry Guided Radiofrequency Hyperthermia. Sensors.

[31] Shi Y., Eberhart R. (1998). A modified particle swarm optimizer. Proceedings of the 1998 IEEE international conference on evolutionary computation proceedings. IEEE world congress on computational intelligence (Cat. No. 98TH8360), Anchorage, AK, USA, 4–9 May 1998.

[32] Lazebnik M., Madsen E.L., Frank G.R., Hagness S.C. (2005). Tissue-mimicking phantom materials for narrowband and ultrawideband microwave applications. Phys. Med. Biol..

